# Theoretical studies of diverse sexual patterns in marine animals

**DOI:** 10.1098/rspb.2022.2229

**Published:** 2023-01-11

**Authors:** Yoh Iwasa, Sachi Yamaguchi

**Affiliations:** ^1^ Department of Biology, Kyushu University, 744 Motooka, Nishi-ku, Fukuoka 819-0395, Japan; ^2^ Institute for Freshwater Biology, Nagano University, 1088 Komaki, Ueda, Nagano 386-0031, Japan; ^3^ Division of Mathematical Sciences, Tokyo Woman's Christian University, 2-6-1 Zempukuji, Suginami-ku, Tokyo 167-8585, Japan

**Keywords:** sex allocation, cost of changes, constraints, enzymatic reaction rates, evolutionary game

## Abstract

Marine animals show diverse and flexible sexual systems. Here, we review several advancements of theoretical studies made in the last decade. (i) Sex change in coral fishes is often accompanied by a long break in reproductive activity. The delay can be shortened by retaining the inactive gonad for the opposite sex. (ii) Barnacles adopt diverse sexual patterns. The game model was analysed assuming that newly settled larvae choose either growth or immediate reproduction and large individuals adjust male–female investments. (iii) Some parasitic barnacles produce larvae with sexual size dimorphism and others produce larvae with the sex determined after settlement on hosts. (iv) In some fish and many reptiles, sex is determined by the temperature experienced as eggs. The dynamics of sex hormones were studied when the enzymatic reaction rates were followed by the Arrhenius equation. The FMF pattern (male at intermediates temperature; female both at high and low temperatures) required some reactions with enhanced temperature dependence at higher temperatures. The game model provides a useful framework for understanding diverse sexual patterns if we incorporate various constraints, such as unpredictability, cost of trait change and social situations. For further developments, we need to consider constraints imposed by physiological and molecular mechanisms.

## 1. Introduction

Before the publication of ‘*On the origin of species*’, Charles Darwin had been working on the morphology and taxonomy of barnacles for 8 years [1,2]. He confirmed many examples of adaptation. This study of barnacles gave him confidence in the concept of ‘natural selection’ as an important process in evolution. Marine animals exhibit diversity and flexibility in the expression of life history and sexual patterns. Sex allocation theory explains them as outcomes of natural and sexual selection [3].

In the last 10 years, the theory has been refined to explain various phenomena. In this review article, we explain several theoretical analyses on the diverse aspects of sexual expression in marine animals. The main mathematical tool adopted was game theory, in which each individual is an agent who chooses its own traits such as the life history, sex expression, breeding sex ratio and body size of their offspring. The theory explains the observed diversity of sex patterns and how they change with different parameters, such as environmental productivity, social situation, future uncertainty, options available to each individual and time and energy costs of changing sexual traits. The pattern may be controlled by physiological, hormonal or enzymatic mechanisms, in addition to natural and sexual selection.

This review article focuses on studies in which simple mathematical models play an important role, rather than providing an overview of all theoretical or computational studies. We start with three examples of theoretical studies on different aspects of sexual systems of marine animals. First, sex change in coral fishes is often accompanied by a long break in reproductive activity. Some species keep the gonad of both sexes with only one active and the other inactive, called the ‘bisexual gonad’. Keeping the inactive gonad for the non-functional sex in the current social situation could shorten the time delay needed for the growth of this gonad after a sudden change in the social situation. However, this is costly to the current reproductive activity. Second, barnacles adopt diverse sexual patterns: hermaphrodites only; dwarf males and large hermaphrodites; or dwarf males and large females. The sexual pattern in the evolutionarily stable strategy (ESS) was analysed with the game model, in which newly settled larvae choose between immediate reproduction as dwarf males and growth to larger size, and large individuals choose male–female investments. Third, some parasitic barnacles produce larvae with clear sexual difference in size, and others produce larvae with the sex determined after their settlement on the host. The model was developed to compare the advantages of an improved chance of encountering suitable hosts and producing sexually dimorphic larvae.

The fourth topic is different from the other three because it emphasizes the role of mechanisms rather than selection. In some fish and many reptiles, sex is determined by the temperature experienced as eggs. Temperature-dependent sex determination was studied by considering the dynamics of sex hormones when the enzymatic reaction rates were followed by the Arrhenius equation of temperature dependence. MF patterns (male–female at low–high temperature) and opposite FM patterns were generated if the sensitivity of enzymatic reactions included in the system are chosen appropriately. However, the FMF pattern (male at intermediate temperature; female both at high and low temperatures), which is reported in many reptiles, required some reactions to have enhanced temperature dependence at higher temperatures than the Arrhenius equation predicts. This conclusion was supported by a recent report of the involvement of a temperature-sensitive ion channel in sex determination.

## 2. Advantage of keeping the inactive gonad—bisexual gonad

The size-advantage model for sex change in coral fishes and some crustaceans is one of the most successful examples of the standard sex allocation theory [3–5]. In the model, each individual should choose whether to reproduce as a male or a female by comparing the expected reproductive success to be gained. Female reproductive success is proportional to the number of eggs produced, which increases with body size. By contrast, the male reproductive success increases with body size, but not as sharply as female success. The optimal strategy for the individual is to be a male if small and a female if large. As an individual grows, it should change sex from female to male. This is the case for the pandalid shrimp [6]. By contrast, in many coral reef fishes, sex change occurs in the opposite direction: small or middle-sized individuals, are females and only the largest individuals is male. In these species, breeding takes place within a territory that is controlled by the harem male that monopolizes siring success by chasing out all competing males. The difference in the direction of sex change is caused by whether males engage in severe fighting.

The size-advantage model can be extended by considering differences in mortality and/or growth rate between sexes [3], which was formalized in terms of dynamic programming [7]. When a male larger than the harem holder joins the mating group, the previous harem holder may change to a female and start producing eggs. Such sex reversal has been reported in several species of coral reef fishes, demonstrated by an experimental manipulation of the social situation [8,9].

The standard theory of sex change can be summarized as follows: each individual takes the sexual phenotype that provides a faster rate of reproductive success in the current social setting, and the sex change takes place when the fitness advantage switches between the two sexual phenotypes.

However, the time required for the sex change can be long. It ranges from a few weeks to several months (reviewed in [10]). During the period of transition, the individual does not gain reproductive success.

One option to shorten the length of time required for sex change is to keep the gonad for the sex that is not used in the current social situation. For example, an individual functions as a female by producing eggs actively, but it may also keep testis in an inactive form (bisexual gonad). Once the social situation changes, a female individual can start male activity with a much shorter time delay if it keeps both an active ovary and inactive testis than if it has only an ovary before the change in social situation. However, keeping the inactive testis while the individual functions as a female is accompanied by a cost. The volume of the ovary would be reduced by the presence of the inactive testis. This decreases the female reproductive success, which is proportional to the number of eggs produced daily. In a similar manner, a male having an active testis and inactive ovary can shorten the time delay if the social situation changes.

### (a) Dynamic optimization model

**Figure 1.**
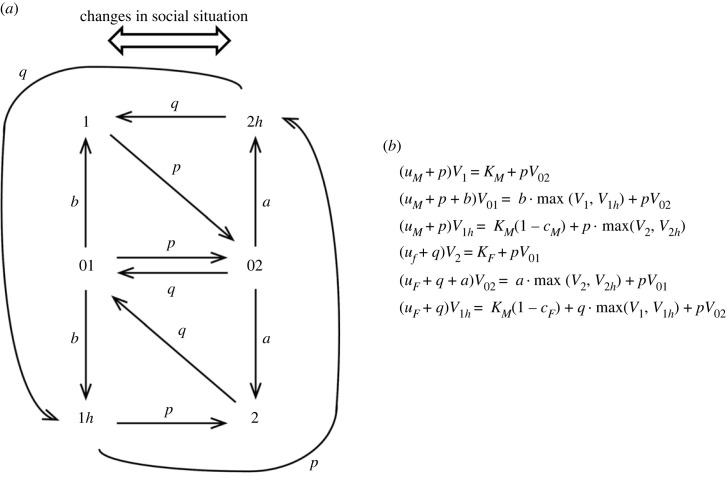
Scheme of the dynamic optimization model for sex changer possibly retaining bisexual gonads. (*a*) Scheme of the model. There are six states (1, 01, 1h, 2, 02 and 2h). The social situation switches randomly following the Markovian process at rates *p* and *q*. (*b*) Equations for future reproductive values held at the stationary state; (*a*) is modified from figure 1 in Yamaguchi & Iwasa [11].

Yamaguchi & Iwasa [11] developed a dynamic programming formalism considering the transition of the social situation and reproductive state of an individual, as illustrated in figure 1*a*. In social situation 1, the individual can act as a harem-holding male, but in social situation 2, it must be a female. The situation is assumed to change randomly in an unpredictable manner at rates *p* and *q* per day, respectively. Consider an individual in situation 1 that actively reproduces as a male. It may have only a testis (i.e. ‘state 1’). The social situation suddenly changes, and the individual needs to become female. Because the individual had only a testis, it should take a sufficiently long time until the maturation of the ovary. This is represented by a non-reproductive period with a mean length of 1/*a*. In the model, this is modelled as ‘state 02.’ Let $V_{1}(t)$ be the expected future reproductive success of an individual in state 1. By considering events in a short period of time Δt, we have the following recursive formula:

2.1 $$V_{1}(t)=K_{M}\Delta t+p\Delta tV_{02}(t+\Delta t)+(1-u_{M}\Delta t-p\Delta t)V_{1}(t+\Delta t).$$

The first term on the right-hand side is the reproductive success as a male to be gained in short period Δt, where *K_M_* is the rate of reproduction as a harem-holding male. The second term is the probability of the change in the social status occurring (pΔt) multiplied by the expected future reproductive success of an individual who is waiting for the maturation of the ovary (state 02). The third term is the probability of nothing occurring multiplied by the expected future reproduction of the male in the same state. Note that there is a chance of death with mortality $u_{M}\Delta t$, which leaves no reproductive success. In the limit Δt→0, we have a differential equation: $dV_{1}/dt=(u_{M}+p)V_{1}-K_{M}-pV_{02}$. The stationary state of this equation is the first line of figure 1*b*.

We now consider the expected future reproduction of an individual in situation 2. The focal individual is a female. However, the ovary is still immature and the individual waits for maturation (state 02). By considering events in a short time interval Δ*t*, we have

2.2 $$\begin{array}{rl} V_{02}(t) & =a\Delta t\cdot \max(V_{2}(t+\Delta t),V_{2h}(t+\Delta t))\\ & \;+q\Delta tV_{01}(t+\Delta t)+(1-u_{F}\Delta t-q\Delta t-a\Delta t)V_{02}(t+\Delta t). \end{array}$$

The mean waiting time until maturation is 1/*a*, which is represented by the fact that in a period of Δ*t*, the individual will shift to the fully functional female at probability *a*Δ*t*. Once the ovary matures, the individual becomes able to function as a female. Then, the individual chooses between being a pure female with only the ovary (state 2) or having both the ovary and testis, the latter being inactive (state 2h). This choice is made by comparing the future reproductive success between the two, which is represented by symbol max in equation (2.2). The second term on the right-hand side of equation (2.2) is for the probability of the social situation changing to situation 1 multiplied by the expected future success in that case. The third term is the probability of the focal individual remaining the same after the interval Δ*t*, i.e. no ovary maturation, no change in social environment and no individual death. In the limit Δt→0, we have the differential equation, and at equilibrium, we derive the fifth equation in figure 1*b*. In a similar manner, we can derive the six equations in figure 1*b* and obtain the solution by solving them.

The equations include $\max(V_{2}(t+\Delta t),V_{2h}(t+\Delta t))$ and $\max(V_{2}(t+\Delta t),V_{2h}(t+\Delta t))$. There are four parameter regions that differ as to which of the two quantities is larger. They are (i) both males and females retain the gonad that is not used in the current situation, where the ‘bisexual gonad’ is retained all the time; (ii) neither males nor females retain the gonads of the currently unused sex. Two other cases are asymmetric between the two sexes: (iii) females retain inactive testes, but males do not retain ovaries and (iv) males retain inactive ovaries, but females do not retain testes.

### (b) Predicted patterns and observations

Yamaguchi & Iwasa [11] examined a few species of sex-changing coral fish that have been studied intensively. In *Trimma okinawae*, both males and females retain the gonad of the unused sex (case 1) and in *Paragobiodon echinocephalus*, neither males nor females retain the gonad of the unused sex (case 2) [14–19]. In both cases, the model's prediction with estimated parameters was consistent with the observed patterns.

There was one parameter that could not be obtained directly from field studies, namely, the cost of retaining the gonad for the sex not currently used. Yamaguchi & Iwasa [11] assumed that the individual retaining the gonad for the unused sex has a reproductive success smaller than the one without it (by a factor of 1−u). To fit this result, Yamaguchi & Iwasa [11] found that the female's cost of having an inactive testis must be more than 20 times greater in magnitude than the male's cost of having an inactive ovary for the model to be consistent with the observed patterns. This conclusion seems plausible because female fitness is equal to the number of eggs produced per day, which would be reduced if some volume of the gonad was occupied by the inactive testis. By contrast, reducing the testis's volume by keeping the unused ovary may not cause much harm to the male's reproductive success because male reproductive success is determined mainly by the ability to chase away competing males.

## 3. Sexual patterns in barnacles and environmental productivity

Barnacles are crustaceans with sedentary adults producing planktonic larvae. In shallow water, most barnacles are hermaphroditic. An individual extends a long penis and copulates with neighbours. Charnov [13] developed the sex allocation theory of barnacles and discussed the ESS ratio of reproductive allocation between two sexes. Yamaguchi *et al*. [20] improved on the Charnov's model by classifying individuals according to their own body size and according to the number and sex ratio of other individuals in the neighbouring area. Numerical analysis of the model explained that in deep water where productivity is low, small individuals start immediate reproduction as dwarf males, and they coexist with large females [20]. Later, the model was extended to consider the case when sperm are used as nutrition [21].

### (a) Choices between reproduction or growth and sex allocation of large individuals

To understand the conditions in which dwarf males occur in the ESS more clearly, Yamaguchi *et al*. [22] developed a simple model with the following assumptions: when a larva is settled on the benthic habitat, it chooses between the two life-history options: either to stay immature and grow larger or to start reproduction immediately as a dwarf male. The choice between the two should be made by comparing their expected future reproductive successes. If large individuals are hermaphroditic, dwarf males must compete with the male function of large individuals for the opportunity to sire eggs produced by the female function of large individuals. Otherwise, the large individuals may be pure females that produce eggs only. Individuals that grow larger suffer a risk of being killed before starting reproduction, which is more important when the growth rate is slow.

**Figure 2.**
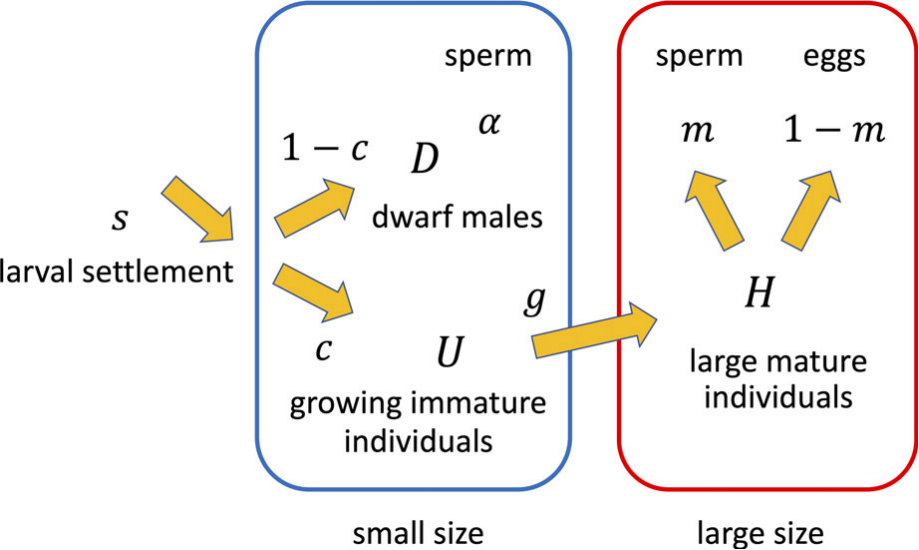
Scheme of the game model for barnacle life history. There is a constant flux of newly settled larvae at rate *s*. Larvae join the left population indicating small individuals. They choose between immediate reproduction as dwarf males (D) or postpone reproduction by aiming to grow (U). The latter become large individuals (H) at rate *g*. Then, they choose the allocation to male and female functions (*m* and 1−m, respectively).

To be specific, Yamaguchi *et al*. [22] studied a model with benthic individuals of just two size classes (small and large) living in habitats such as the shell of a crab as illustrated in figure 2. There is a constant flux of new larval settlement at rate *s*. Fraction *c*(*t*) of these larvae remained immature and grew larger. Growth is represented as a transition from small to large sizes at rate *g*. The remainder of the newly settled larvae (fraction 1−c(t)) immediately start reproduction as dwarf males. The numbers of dwarf males, immature growing individuals and large mature individuals were denoted by *D*(*t*), *U*(*t*) and *H*(*t*), respectively. The differential equations of these population sizes were derived.

The expected future reproductive successes of a dwarf male, a growing immature individual and a large individual were denoted by $V_{D}(t)$, $V_{U}(t)$ and $V_{H}(t)$, respectively. The recursive formula for $V_{D}(t)$ is given as follows:

3.1 $$V_{D}(t)=\alpha *\frac{\left(1-m^{*}(t))H(t\right)}{\alpha D(t)+m^{*}(t)H(t)}\Delta t+(1-u\Delta t-\mu \Delta t)V_{D}(t+\Delta t),$$

The first term on the right-hand side indicates the siring success of a dwarf male. *α* is the ratio of the reproductive contribution by a dwarf male to that by a large individual. It is multiplied by the ratio of the total egg production to the total male activity in the local habitat. A large individual can be hermaphroditic, making both male investment (sperm production) and female investment (egg production) with the allocation ratios, *m* and 1−m, respectively. *α* is likely to be smaller than 1 because a dwarf male is much smaller in size than a large individual. *u* and μ are the mortality of dwarf males and the rate of habitat loss, respectively. From equation (3.1), in the limit Δt→0, the differential equations for $V_{D}$, the expected future reproductive values of a dwarf male were derived as follows:

3.2 $$-\frac{dV_{D}}{dt}=\frac{\alpha (1-m^{*}(t))H(t)}{\alpha D(t)+m^{*}(t)H(t)}-(u+\mu )V_{D}(t)$$

In a similar manner, the differential equations for *V_U_,* and *V_H_* were derived. In total, three differential equations for the population sizes and three more for the future values of three states were derived.

In the ESS, the following two optimization conditions hold

3.3a $$\max_{c}[cV_{U}(t)+(1-c)V_{D}(t)],$$

and

3.3b $$\max_{m}\left[1-m+m*\frac{\left(1-m^{*}(t))H(t\right)}{\alpha D(t)+m^{*}(t)H(t)}\right].$$

Equation (3.3*a*) indicates the choice of newly settled larvae between immediate reproduction as dwarf males or delayed reproduction as growing individuals. Equation (3.3*b*) indicates the choice of large mature individuals concerning the allocation between male and female function.

At time *T*, the host will moult, and all the barnacles on the shell will be discarded, implying that all three future values are zero at t=T. Yamaguchi & Iwasa [22] integrated the differential equations for $V_{D}$*,* $V_{U}$ and $V_{H}$ backward with respect to *t* from *T* to 0 and the differential equations for *D*, *U* and *H* forward with respect to *t* from 0 to *T*. By trial and error, *c*(*t*) and *m*(*t*) satisfying all the necessary conditions were determined. It is an evolutionarily stable sexual pattern [22].

### (b) Constraint for newly settled larvae to become dwarf males

If the growth rate is fast (large *g*), all the newly settled individuals aim to grow larger, and no dwarf male occurs (c* = 1). The large individuals become hermaphrodites with equal investment on both sexes (m* = 1/2). By contrast, if the productivity in the local water is low, the growth rate *g* is small, and a fraction of newly settled individuals start reproduction immediately as dwarf males ($0<c^{*}<1$). The rest of the settled individuals choose to grow. When they become large, all of them become females (m* = 0) because the presence of many dwarf males makes male investment not profitable for large individuals [22].

**Figure 3.**
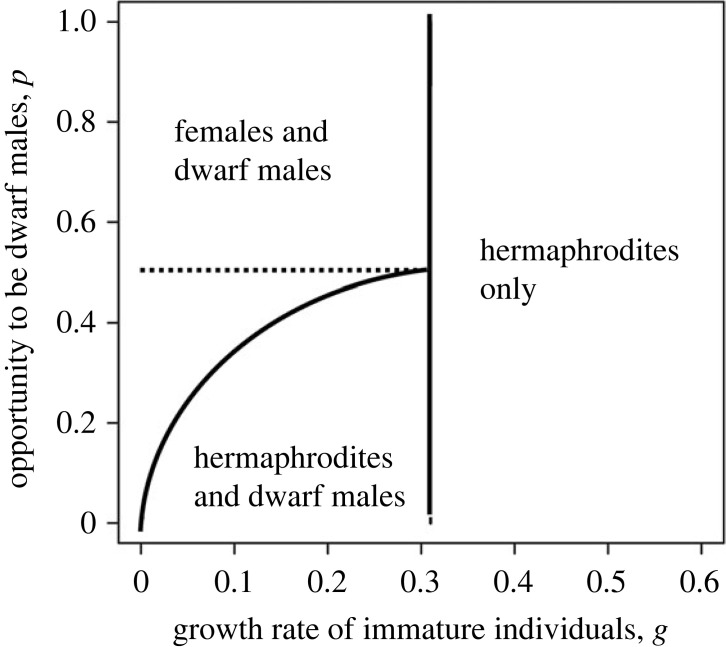
Parameter ranges for different sexual patterns. The horizontal axis is *g*, the growth rate for immature individuals. The vertical axis is *p*, the fraction of newly settled larvae that are allowed to become dwarf males. If growth is fast (large *g*), all individuals grow to large hermaphrodites and no dwarf male occurs. If growth slows, some larvae become dwarf males, and large individuals may be either females or hermaphrodites. The shaded area indicates the parameter region for the coexistence of dwarf males and large hermaphrodites, which occurs only when *p* is small. In some parameter regions, the future reproductive value of a dwarf male is larger than that of a growing immature individual ($V_{D}>V_{U}$), but in other regions they are the same ($V_{D}=V_{U}$). This is modified from figure 1 in Yamaguchi *et al*. [25].

In the ESS of this model, a mixture of hermaphrodites and dwarf males appeared only temporarily in a short period in the ESS and over a narrow parameter range. This result contradicts with a stable mixture of dwarf males and hermaphrodites observed in some barnacle species [23,24]. Yamaguchi *et al*. [25] developed a model considering an additional constraint: a newly settled larva can become a dwarf male only if it encounters large individuals to which it attaches. A similar life-history choice is adopted in the marine worm *Bonellia* [26]. A larva that fails to encounter large individuals must grow itself to a large individual. Figure 3 illustrates the parameter regions in which different sexual patterns evolve. The vertical axis is *p*, the fraction of newly settled larvae that are allowed to become dwarf males.

If the newly settled larvae are free to choose between dwarf males (D) and growing immature individuals (U), the future reproductive success is the same between them at the ESS (*V_D_* = *V_U_*). However, if the availability of becoming dwarf male is limited, the expected fitness of a dwarf male can be larger than that of a growing individual ($V_{D}>V_{U}$). In such a case, becoming a large individual with delayed reproduction is the life-history option considered to make ‘the best of a bad job’ [27].

## 4. Sex determination in parasitic barnacles

Parasitic barnacles infect other crustaceans, such as crabs and hermit crabs [28]. A female parasite manipulates the host and makes it take care of the parasite's eggs. In many species, the female accepts dwarf males in receptacles. Males that succeeded in entering a receptacle keep producing sperm for years [29,30]. Jens Høeg identified two major groups of parasitic barnacles with clear differences in their non-feeding larvae [29]. In species in ‘system 1’, male and female larvae clearly differ in body size. By contrast, in species in ‘system 2’, there is no distinction between male and female larvae and the larvae are suspected to have their sex determined after they settle on hosts.

Sex determination after an encounter with a host improves the chance of encountering suitable hosts: a host not infected by parasitic barnacles is suitable for female larvae to settle but not suitable for male larvae. By contrast, a host already infected by a female is not suitable for female larvae but is very suitable for male larvae. If the larvae are committed to be one sex (either male or female), the likelihood of them encountering a suitable host is smaller than that of larvae that can choose their sex after the host encounter.

Conversely, there exists an advantage for the earlier commitment to a specific larval sex. When producing larvae, the mother chooses the body size and number of larvae. The optimal body size for a larva is determined by the shape of size-fitness curve [31]. If male larvae must engage in severe fighting for the chance of being accepted by a female, the optimal size of male larvae is much larger than that of female larvae. If the mother can decide the sex of each larva while they grow in her body, she can adjust the sizes of the male and female larvae to their respective optimal values. By contrast, if the sex of larvae is determined only after they settle on hosts, the mother must choose the larval size to be an intermediate value that is between the male and female optima, which causes inefficiency of resource use. If the loss in the mother's fitness is larger than the loss of opportunity for larvae to encounter suitable hosts, the mother evolves to produce larvae with predetermined sex, while if it is smaller, the mother evolves to produce larvae with environmental sex determination. Hence the mode of sex determination is closely linked with the need to produce larvae of different sizes, which originates from the intensity of intrasexual competition.

### (a) Evolutionarily stable strategy larval size and sex determination

This scenario was analysed by a game model [32]. First, consider a mother producing male and female larvae with sizes *x* and *y*, respectively. The total amount of reproductive resources is ρ and the allocation ratio to males is *r*. The mother's reproductive success is given as follows:

4.1a $$\phi (r,x,y)=\frac{\rho r}{x}O_{m}S_{m}(x)R_{m}+\frac{\rho (1-r)}{y}O_{f}S_{f}(y)R_{f}.$$

The first and second terms on the right-hand side of equation (4.1a) are the mother's reproductive success through her sons and daughters, respectively. ρr is the reproductive resources used for producing male larvae and ρr/x is the number of male larvae. *O_m_*, *S_m_*(*x*) and *R_m_* are the opportunity for encountering suitable hosts, winning probability and reproductive success of a winning male, respectively. The winning probability for a male larva is an S-shaped function of size *x*, such as $S_{m}(x)=S_{m0}\exp(-a_{m}/x)$. The factors included in the second term can be interpreted in a similar manner. From the condition that the total reproductive success by all the males in the population and that by all the females are equal, we can derive that the ESS value of the sex allocation ratio is r* = 1/2 [32]. The optimal sizes of male larvae and of female larvae are *x* maximizing *S_m_*(*x*)/*x*, and *y* maximizing *S_f_*(*y*)/*y*, respectively.

When a mother produces larvae with environmental sex determination, the mother's reproductive success is given as follows:

4.1b $$\phi_{\mathrm{ESD}}(z)=\frac{\rho }{z}({\bar{O}}_{m}S_{m}(z){\bar{R}}_{m}+{\bar{O}}_{f}S_{f}(z){\bar{R}}_{f})(1-d),$$

where the body size of larvae *z* is a single quantity. The meanings of the symbols with bars are similar to those without bars in equation (4.1a) [32]. The opportunity to encounter suitable hosts is higher than when the sex of larvae is determined, which improves the opportunity to encounter with suitable hosts. Conversely, the delay in development after settlement would cause a disadvantage in fighting larvae of predetermined sex. In equation (4.1b), this is represented by 1−d, a factor smaller than 1.

We first calculate the optimal sizes of male and female larvae (*x* and *y*) maximizing equation (4.1a) and the optimal size (*z*) maximizing equation (4.1b) separately. Then we compare the mother's reproductive success between the two cases: $\phi (1/2,x^{*},y^{*})$ and $\phi_{ESD}(z^{*})$. The one that achieves the highest is the ESS solution.

**Figure 4.**
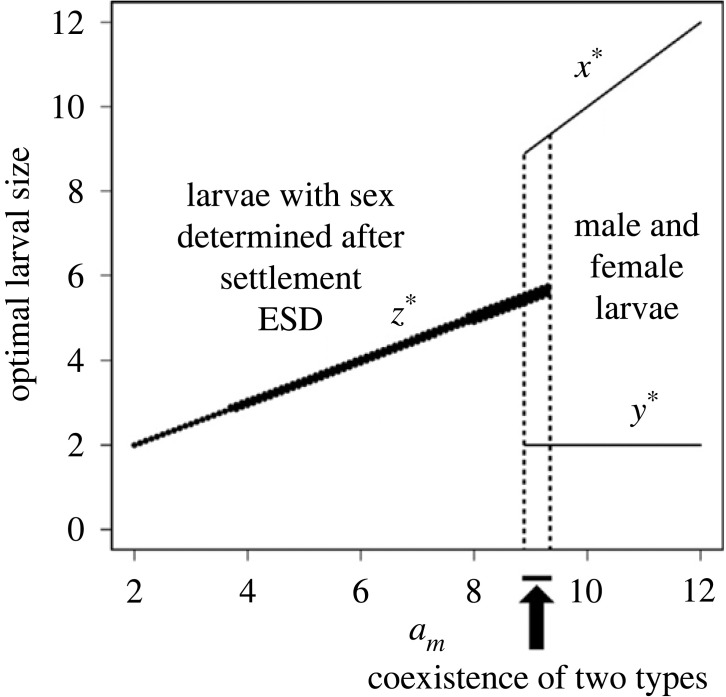
ESS larval sizes for parasitic barnacles. The horizontal axis indicates parameter *a_m_* for the intensity of male–male competition. For small *a_m_*, the mother produces the larvae without a sexual difference. Their sex is determined after they settle on hosts. For large *a_m_*, the mother produces male and female larvae with larger sizes for males. This is modified from figure 5 in Yamaguchi *et al*. [32].

Figure 4 illustrates the optimal larval sizes, where the horizontal axis represents a parameter *a_m_* indicating the intensity of male–male competition. If male–male competition is weak, the difference in the optimal sizes of male and female larvae is rather small, and the improved opportunity of encountering a suitable host by producing larvae of an intermediate size with environmental sex determination should evolve (system 2). If male–male competition is strong, the advantage of producing larvae of sexually different sizes between sexes is more important than the reduced chance of encountering suitable hosts, and larvae of predetermined sex should evolve (system 1).

The difference between the two systems originates from the difference in the female mating behaviour. In the species of system 1, a female accepts only two males in her receptacles and then does not accept more males. This leads to very intense competition between the larvae, which results in a large optimal size for a male. In species of system 2, a female accepts many males that can become implanted and contribute to reproduction, which leads to a milder competition among males [32].

## 5. Temperature-dependent sex determination controlled by enzymatic reactions following the Arrhenius equation

In some fishes (e.g. Atlantic silverside; [33,34] and many species of reptiles [12,35–38]), sex is determined by the temperature experienced in the egg stage. There are several different patterns: eggs develop to males at low temperatures but to females at high temperatures in some species [39]. The opposite temperature dependence is adopted in other species [38,40]. In the American alligator *Alligator mississippiensis* [37] and snapping turtle *Chelydra serpentina* [35,41], eggs develop to males at an intermediate temperature range and to females at both high and low temperatures. We may call these the MF pattern, FM pattern and FMF pattern, respectively [42]. Among them, the FMF pattern is suspected to be the original pattern [40,43]. Interestingly there is no report of MFM pattern in which the eggs develop to females at intermediate temperature and to males at both high and low temperatures.

In some species, the temperature-dependent sex determination patterns have been proven to be adaptive [44]. In the short-lived lizard *Amphibolurus muricatus*, eggs develop to females if they experience a high incubation temperature and to males if they experience a low temperature. A warm hatching environment provides faster growth, which leads to a larger body size with enhanced reproductive success. The correlation between reproductive success and body size is stronger in females than in males. Hence, when eggs are incubated in a warm local habitat, they will enjoy higher fitness by developing to females than developing to males. By contrast, when eggs are incubated in a cold habitat, their fitness is improved by developing to males. The logical steps of this explanation have been confirmed in field studies over many years [45].

Interestingly, the opposite relationship between sex and temperature was reported for Atlantic silverside [33]: a cold hatching temperature of this fish produces females and a warm hatching temperature produces males. For this species, hatching temperature is correlated with the seasonal timing of egg production. A cold hatching temperature indicates that it is early summer and a long time until the breeding season, so developing to female is advantageous. By contrast, a warm hatching temperature indicates that it is midsummer, suggesting a shorter period of time for growth until the breeding season, which makes developing to males is advantageous [34].

**Figure 5.**
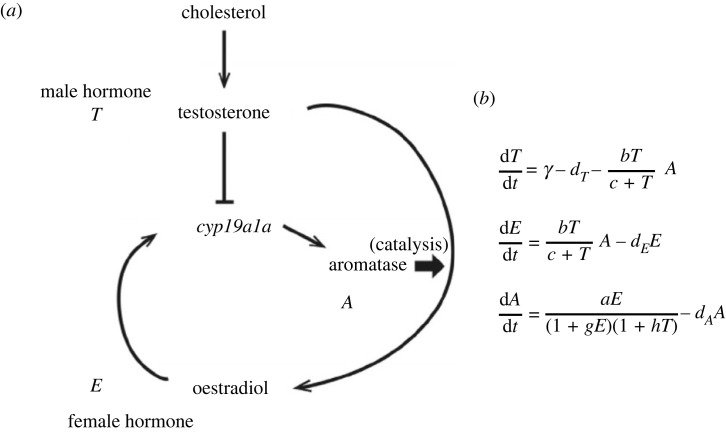
A simplified dynamics of sexual hormones. (*a*) Scheme of the dynamics. Testosterone and oestradiol are male and female hormones, respectively. Oestradiol is formed from testosterone by the action of the enzyme aromatase. Aromatase production is activated by oestradiol and suppressed by the abundance of testosterone. This is modified from figure 1 of Yamaguchi & Iwasa [42]. (*b*) Simple dynamics of enzymatic reactions *T*, *E*, *A* are testosterone, oestradiol and aromatase, respectively. γ, *a*, *b*, *d_T_*, *d_E_* and *d_A_* indicate reaction rates, and they increase with temperature.

Yamaguchi & Iwasa [42] developed a theoretical model for the temperature sex determination controlled by physiological or enzymatic mechanisms. They noted that the female sex hormone oestradiol is produced from the male sex hormone testosterone by the action of an enzyme called aromatase, as illustrated in a simplified scheme in figure 5*a*. The eggs develop to males if testosterone is abundant and to females if oestradiol is abundant. An example of the simple dynamics of these hormones is given in figure 5*b*. The rate of each reaction increases with temperature because all enzymatic reactions occur faster at a higher temperature unless the enzyme undergoes denaturation. If all the enzymatic reactions become twice as fast as before, the equilibrium abundance of the two sex hormones (testosterone and oestradiol) remains the same. However, temperature sensitivity may differ between enzymes. Some enzymatic reactions may occur much faster at a higher temperature than at a lower temperature, while the rate of other enzymatic reactions may increase with temperature only slightly. This may produce a situation in which at a low temperature, the stationary level of oestradiol is high and that of testosterone is low, but at a high temperature, that of oestradiol is low and that of testosterone is high. This implies that the system shows an FM pattern. In other cases, the stationary level of oestradiol is high and of testosterone is low for low temperature, while the opposite occurs for high temperature, which corresponds to the MF pattern.

Yamaguchi & Iwasa [42] considered the situation in which the enzymatic reactions follow the Arrhenius equation: the rate is proportional to exp[−ΔE/kT], where ΔE is the activation energy, *k* is the ideal gas constant and *T* is the absolute (Kelvin) temperature. This rule is established for many catalytic reactions, and it is also justified by diffusion theory [46]. The logarithm of the enzymatic reaction rate and the inverse of absolute temperature (1/*T*) are plotted on a plane, the data should be on a straight line if the Arrhenius equation holds.

**Figure 6.**
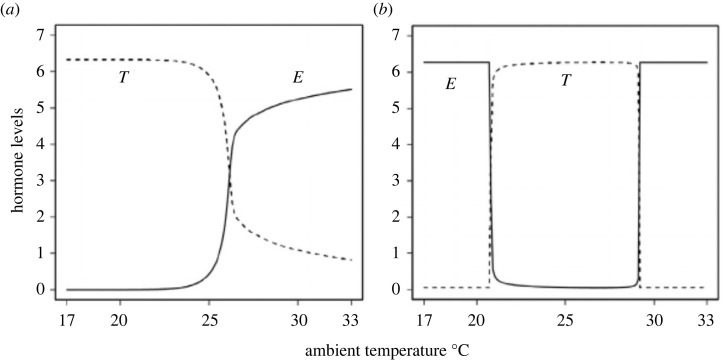
Patterns of temperature-dependent sex determination. The vertical axis indicates the abundance of two sex hormones (testosterone and oestradiol), and the horizontal axis represents temperature in Celsius. (*a*) MF pattern. This is from figure 2*b* in Yamaguchi & Iwasa [42]. The aromatase reaction rate *b* has a strong temperature dependence, but all the other reactions have a weak temperature dependence. (*b*) FMF pattern. This is from figure 6*a* of Yamaguchi & Iwasa [42]. The aromatase decay rate *d_A_* followed the Arrhenius equation, but the aromatase reaction rate *b* followed the Berthelot equation, the latter indicating stronger temperature sensitivity at high temperatures than the Arrhenius equation.

Yamaguchi & Iwasa [42] examined numerous combinations of parameters, such as standard reaction rate and temperature sensitivity for all the reactions included in the system, as shown in figure 5. For each combination, they examined the levels of two sex hormones at equilibrium for temperatures from 17°C to 33°C in increments of 0.5°C. In some choice of parameters, as illustrated in figure 6*a*, the testosterone concentration at equilibrium was high below a temperature but high above it but the oestradiol concentration was low below it and high above it, and the oestradiol concentration changed sharply within 0.5°C (more than one-third of the difference between the maximum and the minimum). This pattern was interpreted as realizing an MF pattern of temperature dependence. An exhaustive examination of $3^{9}=19683$ parameter combinations revealed that MF patterns were produced if *a* and *b* were strongly temperature-sensitive but *d_A_* and *d_E_* were not. In the opposite situation (*d_A_* and *d_E_* were strongly temperature-sensitive but *a* and *b* were not, FM patterns were produced.

Interestingly, there was no case in which the FMF pattern of temperature sensitivity was adopted in the snapping turtle *C. serpentina* [47] and alligator *A. mississippiensis* [40].

The Berthelot equation is a rule for the temperature dependence of enzymatic reactions different from the Arrhenius equation. It postulates that the rate is proportional to an exponential function of temperature (e.g. exp(*CT*)), which has a stronger temperature dependence for a higher temperature compared with the Arrhenius equation. Yamaguchi & Iwasa [42] examined the situations in which one of the enzymatic reactions follows the Berthelot-Hood equation but all the other reactions follow the Arrhenius equation. As shown in figure 6*b*, the FMF pattern can be produced if either the production rate or the reaction rate of aromatase (*a* or *b*) follows Berthelot-Hood equation, while other rates follow the Arrhenius equation. By contrast, an MFM pattern was produced if either *d_A_* or *d_o_* followed the Berthelot-Hood equation, but others followed the Arrhenius equation. Because the FMF pattern of temperature dependence is adopted in many reptiles, but MFM pattern is not reported, Yamaguchi & Iwasa [42] concluded that the production or reaction of aromatase (i.e. *a* or *b*) should include a mechanism making the rate more strongly temperature-dependent at high temperatures than indicated by the Arrhenius equation (such as temperature-dependent splicing). Yatsu *et al*. [48] revealed that a temperature-sensitive ion channel, named TRPV4, is involved in sex determination in *A. mississippiensis*. Their study shows that the Arrhenius plot of TRPV4 appears not as a straight line but rather as two lines with a steeper slope for a higher temperature than for a lower temperature (M Tominaga 2022, personal communication), as is common to a similar temperature-sensitive ion channel TRPA1 [49]. This is consistent with the results expected by Yamaguchi & Iwasa [42].

## 6. Discussion

Sex allocation theory postulates that the evolutionary endpoint is the state in which each individual attempts to maximize its own reproductive success or inclusive fitness chosen among a range of phenotypes that are available for the focal individuals [3]. Because of the conflict of interest among players, the model may not be a simple optimization but rather a game.

We reviewed several recent advancements in theoretical studies on the sex allocation of marine animals. The first three were the extensions of the basic sex allocation theory considering the constraints that had not been emphasized before.

The first example illustrated the importance of the cost of phenotypic changes. In coral fishes, after a change in social situation, sex change often takes a long time until reproductive activity [10]. The delay can be shortened if the inactive gonad for the sex not used in the current social situation is retained at the cost of a reduced volume of the gonad for the currently active sex. A dynamic programming model applied to well-studied species demonstrated that the cost for the female to retain inactive testis was much larger than that for the male to retain an inactive ovary. This is plausible because female reproductive success increases with the egg production rate, but male reproductive success is determined by the ability to chase away competing males rather than by the sperm production rate.

The second example was about the constraint of life-history choice. To understand the diversity of sexual patterns in barnacles, a dynamic programming model was studied in which (i) newly settled larvae choose between immediate reproduction as dwarf males and delayed reproduction by growing to a large size and (ii) large individuals choose sex allocation between male and female functions. In productive environments, barnacles are hermaphroditic, while in less productive environments such as the deep sea, they show a combination of dwarf males and large females. The combination of dwarf males and large hermaphrodites was observed in many species of barnacles [23], but it was not the ESS if the newly settled larvae were free to choose to be dwarf males. It is the ESS if only a small fraction of newly settled larvae can be dwarf males, which is biologically plausible [26,50,51].

The third example showed that the adaptation of sex determination is linked with many other aspects in the larval life history, such as the size difference in larvae, male–male competition, and number of males accepted by a female. In parasitic barnacles infecting crabs and hermit crabs, females infect hosts and control host behaviour. If males are accepted by a female, they stay as dwarf males and keep producing sperm for many years. Parasitic barnacles consist of two distinct groups: In system 1, the mother produces larvae with a clear difference in size between the sexes. In system 2, the mother produces larvae whose sex is determined after they settle on hosts. The trade-off between these two distinct types is determined by relative advantage of having larvae of sexually different sizes between the sexes and the improved opportunity of host encounter.

The last example was different from others because it concerns the enzymatic mechanism that realizes the phenotypic change in response to the environment. In some fish and many reptiles, sex is determined by the temperature experienced in the egg stage. The dynamics of male/female hormones were examined, in which the enzymatic reactions increased with temperature following the Arrhenius equation. The MF pattern (male at low temperature and female at high temperature) and the opposite FM pattern could be explained if the temperature sensitivity of the reactions were chosen appropriately. However, the FMF pattern (male at the intermediate temperature range and female both at high and low temperatures) was not possible to generate. This suggests that some enzymatic reactions may have a stronger temperature dependence at high temperatures than indicated by the Arrhenius equation. This is consistent with a report that the temperature-sensitive ion channel TRPV4 is involved in the sex determination of some reptiles [48]. In a different species, the Chinese alligator, a different ion channel seems to be adopted to produce sex differences [52]. There are attempts to determine the underlying mechanisms that realize the temperature-dependent sex determination, such for different species of snapping turtle [53]. We hope further studies on the physiological and molecular mechanisms will clarify this issue in the near future.

A similar way of thinking is useful in clarifying other aspects of life history of marine animals. In the following section, we discuss just two examples below.

### (a) Androdioecy—rare sex expression in the animal kingdom

In the animal kingdom, androdioecy (the coexistence of hermaphrodites and males) is very rare. It has been reported in barnacles, nematodes such as *Caenorhabditis elegans*, clam shrimps, tadpole shrimps and mangrove killifishes [54]. Barnacles are different from others because hermaphrodites of barnacles can undergo outcrossing. In all the other androdioecious species, hermaphrodites reproduce by selfing and engage in outcrossing only with males [55]. Offspring produced by selfing have lower fitness than those produced by outcrossing, because of inbreeding depression [56]. The evolutionary stable ratio of different sexual phenotypes critically depends on the magnitude of inbreeding depression. In both clam shrimps and tadpole shrimps, chromosomal sex determination has been identified from genomic studies, and an evolutionary genetic theory on genetic control was developed [57]. By contrast, mangrove killifishes exhibit flexible sex determination depending on the temperature and pH in the environment [58,59]. The reported fraction of males is positive and it varies between local populations, but it is much lower than the prediction of simple models. Yamaguchi & Iwasa [60] developed a theoretical modelling of the sexual system of killifish to explore possible population structures, but full understanding requires more empirical studies.

### (b) Diversity of life cycle patterns

In many marine invertebrates, adults are sessile and benthic. They produce planktonic larvae, which grow and experience several steps of metamorphosis, and then settle on the benthic habitat. Their life cycle has two different modes of acquiring food resources (benthos and plankton), which is called the complex life cycle. In high latitudes and the deep sea, some marine animals have life cycles of direct development, which skip the planktonic larval stages. Their benthic adults produce small benthic offspring in the same local habitat [61].

To study the evolution of life cycle types, Yamaguchi *et al*. [62] considered the biomass dynamics of two types: the larvae of the complex life cycle type are planktonic during summer months, settle onshore in the autumn and spend winter months as benthos, while the direct development type is benthic year-round. The competitive ability during winter months was assumed to be the same between the two. The outcome of their competition was as follows: (i) only a complex life cycle remained, (ii) only a direct development type remained or (iii) the two life cycle types coexisted, depending on parameter values. In the field, the coexistence of two life cycle types in a single species is very rare. The only clear example is sea slugs (Sacoglossa) [63], which perform kleptoplasty, keeping chloroplasts from algal food in their body for a limited length of time [63,64]. They use the photosynthetic products of the chloroplasts as nutrition. Their algal food is strongly limited taxonomically and geographically, which is consistent with the model's prediction that a stable coexistence of life cycle types is possible only when resource competition is intense [62].

There exists a third life cycle type called lecithotropy. Species with lecithotropy have planktonic larvae that do not obtain food from the environment but live on the resources given by their mother [65]. After a short planktonic stage, they settle on the benthic habitat. In terms of biomass acquisition, lecithotropy is less efficient than direct development due to the loss in the non-feeding planktonic stage. However, having a short planktonic stage allows dispersal between local benthic habitats. Iwasa *et al*. [66] considered the situations in which the habitat for benthic individuals consists of many local patches that receive disturbances in a manner not synchronized. Iwasa *et al*. [66] also considered limited migration of adults just before reproduction. They identified the conditions in which one of the three life cycle types (direct development, benthos and feeding plankton, benthos and non-feeding plankton) evolves, as well as parameter ranges in which multiple life cycle types coexist.

From this review, we conclude that the game models of sex allocation are useful for understanding diverse sexual patterns in general, but we need to incorporate various constraints, such as unpredictable environmental changes, cost of trait shifts and social constraints. In addition, we believe that further development of the topics must incorporate not only fitness maximization but also constraints imposed by mechanisms that have been clarified by recent advancements in life science, such as physiological, hormonal, enzymatic, epigenetic, genetic and molecular studies.

## Data Availability

This article has no additional data.
